# Corneal transplantation during COVID-19 pandemic: need for special considerations—A live review

**DOI:** 10.3934/publichealth.2021014

**Published:** 2021-02-24

**Authors:** Anuj Kumar Pandey, Nidhi Mudgil, Yogesh Wadgave, Sidharth Sekhar Mishra

**Affiliations:** Disaster Management Cell, Ministry of Health and Family Welfare (MOHFW), India

**Keywords:** corneal transplantation, COVID-19, corneal harvesting, eye banking

## Abstract

Corneal blindness is the fourth leading cause of blindness worldwide, with 10 million people having bilateral corneal blindness, nearly 80% of all corneal blindness cases are avoidable and are reversible. Corneal transplantation (CT) is the most frequently performed type of transplant across the world. This review was conducted with the objective of identifying if it is safe to harvest the cornea from the patients died due to COVID-19 and preventing the chances of transmission from donor to the recipient or healthcare worker handling the harvested cornea. A total of 45 articles were found with the keywords and out of all, only 16 fulfilled the inclusion criteria. RT-PCR is the technique of choice for detection of virus in the corpse and the sample analyzed was a pharyngeal swab. Available literature states unavailability of sufficient evidence-based studies proving presence of virus in the cornea or tear of COVID affected patients There is no proven consensus on presence of Virus in cornea. It is important to follow preferred practice guidelines so as to restart eye banking and do at least the emergency surgeries without having risk of disease transmission and keeping ourselves safe.

## 1. Introduction

Corneal blindness is the fourth leading cause of blindness worldwide, with 10 million people having bilateral corneal blindness, nearly 80% of all corneal blindness cases are avoidable and are reversible [1].

Corneal transplantation (CT) is the frequently performed type of transplant across the world. It restores visual function when impairment caused by corneal damage deemed too severe to provide acceptable vision and quality of life [1]. CT is most successful organ transplantation in the human body as cornea is devoid of vasculature, minimizing the risk of graft rejection [2]. Till date, keratoplasty is the most commonly performed allogenic transplant worldwide, with a success rate of 69–95% [3],[4]. With an estimated 12.7 million people expecting a corneal transplantation, 1 in 70 of the requirements is covered worldwide. Fifty-five percent (55%) of all cornea gets procured in the United States of America (USA) and India [1].

As per WHO estimates the blind population will double by 2020 due to rise in population and longevity [5]. There are 45 million blind persons in the World, of which 12 million blind persons are in India including 1% of which is due to corneal blindness [5]. In India, corneal related vision loss in one eye is approximately 6.8 million people; of these, about a million have bilateral involvement [5],[6]. It is estimated that approximately 20,000 patients with corneal blindness are added to the backlog each year and that 50% of corneal blindness are treatable [6].

In 1983, the National Health Policy of India stated that blindness was an important public health concern and set a target to reduce it from 1.4% to 0.5% by the year 2010. The Government of India (GoI) has now laid down a target for reduction in the prevalence of blindness to 0.25/1000 by 2025 and disease burden by one third from current levels [7].

The outbreak of Novel Coronavirus Disease (COVID-19) was initially noticed as an outbreak of pneumonia of unknown etiology in Wuhan city in Hubei Province of China in mid-December 2019. The outbreak was found to be caused by a novel Coronavirus (named SARS-CoV-2) on 7^th^ January 2020. It has since spread worldwide. WHO (under International Health Regulations) has declared this outbreak as a “Public Health Emergency of International Concern” (PHEIC) on 30^th^ January 2020. WHO subsequently declared COVID-19 a pandemic on 11^th^ March, 2020. As on 28^th^ January 2021, a total of 100,455,529 confirmed cases and 2,166,440 deaths have been reported globally. Maximum number of cases is currently being reported from USA, India, Brazil, Russia, UK, France, Spain, Italy, Turkey and Germany. A resurgence of cases has been reported from a number of countries in Europe, Americas, Africa and Asia Pacific [8].

In India a total of 10,701,193 positive cases and 173,740 deaths have been reported so far as on 28^th^ January, 2021. In absolute numbers India has reported second highest number of cases and third highest number of deaths globally. However, the country has reported 7655 cases and 110 deaths per million population, which is considerably low than similarly affected countries. As on 28th January 2021, a large majority of total cases (96.93%) have recovered. India's case fatality rate of 1.4% is also lowest among similarly affected countries. As of now, ten States (Kerala, Maharashtra, Uttar Pradesh, Karnataka, west Bengal, Tamil Nadu, Chhattisgarh, Gujarat, Madhya Pradesh, Telangana) are contributing to 88.96% of all active cases in the country. The trajectory of daily number of new cases and deaths continues to show a downward trend since reaching its peak in mid-September 2020 [9].

Various infection control measures have evolved in the last few months to minimize the spread of infection to both patients and health care workers. Hospitals have initiated a triage system to minimize cross-infection. Elective surgeries in all specialties, including ophthalmology, have been suspended since the last week of March 2020 in India due to implementation of nationwide lockdown from March 21^st^, 2020. Ministry of Health and Family Welfare (MoHFW) has issued a guideline stating that, hospitals can continue emergency services following social distancing norms and with all personal protective gears except in containment zones. Elective and non-emergency follow ups and surgeries can be deferred [9].

Several aspects of healthcare including eye donation and eye collection require adequate safety precautions in place to keep both the healthcare workers and patients safe [10]. It has made us change our approach to handle and manage the cases and in the aftermath of this pandemic, ophthalmologists will require practical guidelines based on advisories from national health departments on how to restart eye banking and cornea-related healthcare across the country [11].

A significant issue at present is the interaction of the cornea donors with SARS-CoV-2. Even with our currently limited testing capacity, the confirmed cases across globe numbers are significant, and trending towards an unknown peak [12]. The deaths directly caused by COVID-19, it is expected that a significant number of individuals dying from all other causes will be infected by or exposed to COVID-19. It is therefore probable that a sizable fraction of donated corneas will soon meet a donation exclusion parameter set out by a tissue banking governing body [2].

Majority of the corneal blind patients may be visually rehabilitated by corneal transplantation. An efficient and precise utilization of the donor corneas in developing countries like India is merely possible after an entire knowledge of the collection, storage, and utilization of donor tissues. Thus, this review was conducted with the objective of identifying if it is safe to harvest the cornea from the patients died due to COVID-19 and preventing the chances of transmission from donor to the recipient or healthcare worker handling the harvested cornea.

## 2. Methods

A review of Corneal harvesting from COVID-19 positive dead bodies was conducted based on the reports and articles available in PubMed and ScienceDirect.com. Keywords used were- ((Corneal Harvesting)) and (COVID patients) OR (eye banking during COVID-19) OR (Corneal Transplant) OR (Keratoplasty).

Articles referring to Mode of Transmission of COVID-19 were also included. Articles were searched in the Third week of November 2020.

Articles were assessed based on the criteria to be included for the review:

- Articles/Guidelines/Notes etc. published in last one year.
- Articles related to Harvesting Cornea from COVID-19 affected patients.
- Articles with risk of transmission while harvesting Cornea from COVID affected patients.
- Articles related to eye-banking during COVID times were only included in the study.

## 3. Results

A total of 45 articles were found with the keywords and out of all, only 9 fulfilled the inclusion criteria. In addition to that, Four Guidelines/Technical advisories were included which provided the viewpoints pertaining to cornea Harvesting. Three other articles were also included that were obtained from the references of the identified articles.

Out of nine articles, 5 are editorials/commentary/consensus statement on guidelines for cornea and eye banking during COVID-19, 4 articles are literature reviews. Selection criteria is explained in Flowchart. All these articles have been published in 2020.

A total of 16 articles/Guidelines following the inclusion criteria were reviewed. Details of which is attached in Table 1.

**Figure 1.**
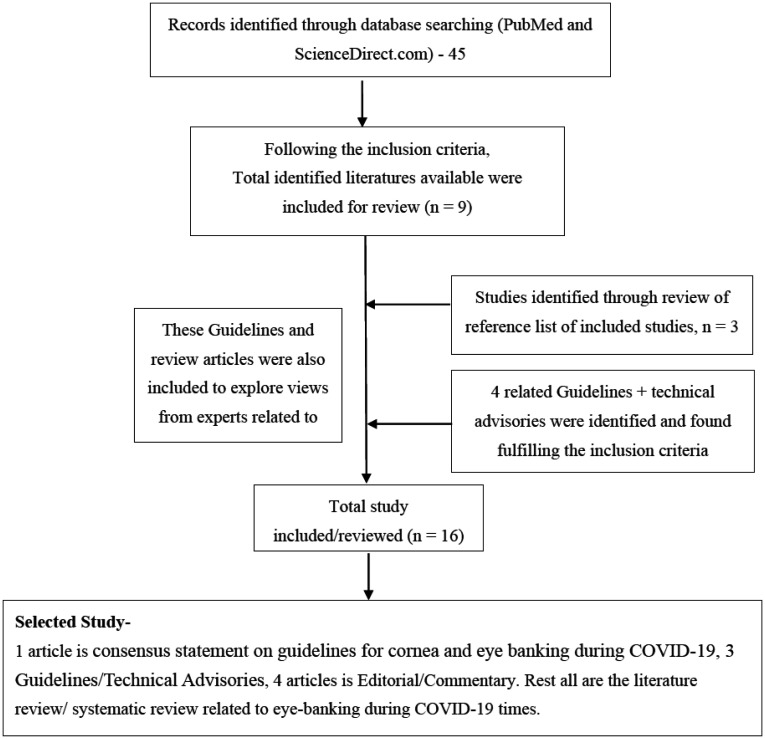
Selection of articles for review.

**Table 1.** Summary of articles on corneal transplantation during COVID pandemic.

Sr No	Author Name	Methodology	Results/Recommendations
1.	Sawant O.B. et al. [13]	n = 33 surgical-intended donors	Of 132 ocular tissues from 33 surgical-intended donors, the positivity rate for SARS-CoV-2 RNA was 13% (17/132). Study recommends post-mortem nasopharyngeal PCR testing and PVP-I disinfection protocol to eliminate any tissue harboring SARS-CoV-2 being used for corneal transplantation.
2.	Roy A. et al. [14]	Descriptive study of the challenges faced in eye banking during lockdown, and practices adopted to overcome	Immediate drastic reduction of donor retrieval. Shift to glycerol preservation. Cessation of precut tissues for posterior lamellar surgeries. Resumption of donor cornea retrieval guidelines. Patient triage for keratoplasty and alternatives to emergency keratoplasty.
3.	Desautels JD et al. [11]	Review of recommendations made by various health agencies/authorities for retrieving Cornea.	Study recommends povidone-iodine does not accomplish complete sterilization, and by no means it eliminates the possibility of viral retention within ocular structures. Infection of deeper cellular layers of corneal donor tissue remains a possibility, and warrants additional study.
4.	Fernández-Rodríguez A et al. [15]	Review of microbiological aspects of COVID-19 infection	No Study reports use of more than one type of sample for microbiological analysis No specific tests are being recommended to use in corpses. Samples to be taken prior to autopsy, more specifically NPS and pharyngeal swab.
5.	Global Alliance of Eye Banking Association [16].	Global committee (GAEBA) alert about Coronavirus (COVID-2019) and Ocular Tissue Donation	Risk to donor ocular tissue is considered low. There have been no reported cases of transmission of SARS-CoV-2, MERS-CoV, or any other coronavirus via transplantation of human ocular tissue. A recent study reported that no SARSCoV-2-RNA was detected in the cornea, conjunctiva, or aqueous humor of five COVID-19 positive post-mortem donors.
6.	Welsh Government COVID-19 Technical Advisory [17].	Technical Advisory	No data is currently available on the frequency of detection of SARS-CoV-2, by RT-PCR on postmortem swabs collected at different durations after death. If COVID-19 testing on postmortem swab specimens is done, SARS-CoV-2 RNA may still be detected up to 3 days postmortem and possibly longer based on available data from experiences with MERS-CoV and SARS-CoV; however, sensitivity may be reduced with a longer postmortem interval, and duration of illness may need to be considered in interpreting a negative result. Thus, in cases where there is a clinical indication for diagnostic testing, samples may be taken within 3 days of death for RT-PCR testing with a reasonable likelihood of detecting virus if present.
7.	Guidelines on postmortem testing [18]	Guidelines on Postmortem Testing for Natural Deaths by Department of Health republic of South Sudan	A single postmortem nasopharyngeal swab (NP swab) is preferred. When the collection of a postmortem NP swab is not possible, an oropharyngeal (OP) specimen, A nasal mid-turbinate (NMT) swab, an anterior naris (nasal swab; NS) specimen, Nasopharyngeal wash/aspirate, or nasal aspirate (NA) specimen will be preferred.
8.	Ang M, Moriyama A et al. [19]	None	Eye Bank Association of America (EBAA) and the Global Alliance of Eye Bank Associations (GAEBA) have recommended excluding donors recently infected with COVID-19, or those at high-risk such as a significant contact history. US Food and Drug Administration (FDA) indicate that there is currently no evidence for transmission of respiratory viruses through tissue transplantation in general. Eye Bank Association of Australia & New Zealand (EBAANZ) and European Centre of Disease Control advises against routine testing for donors due to limited test kits, which are also not validated for use in deceased patients. Further studies on the validity of SARSCoV-2 PCR tests on deceased donors are needed to inform policy decisions on donor testing requirements.
9.	Toro M et al. [3]	Commentary	Study did not find any evidence to substantiate that harvested corneal grafts from COVID-19 patients can contain SARS-CoV-2 virus and lead to a systemic infection. Although risk of transmission through corneal stromal tissue is low, but it potentially exists. Study recommends that the blood samples of all donors should be screened with RT-PCR tests and nasopharyngeal swabs should be taken.
10.	Amesty MA et al. [20]	Review	Study found that although there is a low prevalence of SARS-CoV2 in tears, it is possible to transmit the disease through ocular secretions. The relationship between COVID-19 and the ocular surface as a potential portal of entry and as a transmission mechanism is currently under discussion due to the high transmission rate of the disease.
11.	Chaurasia S et al. [21]	Editorial	Donor cornea, however, carries the unknown and unquantifiable risk of transmission of disease to the recipient. Harvesting eyes from donors with conditions potentially hazardous to eye bank personnel should be strictly avoided. Therefore, death due to COVID-19 will fall into this category. The second category of contraindication includes conditions with the potential risk of transmission of local or systemic communicable disease from donor to recipient. Since there is a risk of transmission via ocular surface, it carries a risk of transmission to the recipient. It is too early to have evidence that SARS-CoV-2 can be transmitted by blood transfusion or tissue transplantation.
12.	Ballouz et al. [22]	Review	There is evidence that transmission through blood donation and organ or tissue transplantation is possible. SARS-CoV-2 has been detected in conjunctival swabs of infected patients, and the ocular surface may play an important role in viral entry. The risk of SARS-CoV-2 transmission through corneal transplantation is likely low. However, tissue screening guidelines need to be re-evaluated regularly as knowledge regarding the SARS-CoV-2 virus evolves
13	Kates OS et al. [23]	Review	Study found that the current data provide little evidence to suggest the presence of intact transmissible SARS-CoV in organs that can potentially be transplanted.
14	Mukhra R et al. [24]	Review	Till date, there is a single report of conjunctivitis along with the viral RNA in the tear body fluid. However, an absence of the virus in the patients' conjunctival sac implicated an unusual route of transmission of SARS-CoV-2. Study recommends more research to develop a detailed understanding of the transmission mechanism through tear secretions and ocular surfaces.
15	Sharma N et al. [25]	None	Paper highlights the consensus-based guidelines by an expert panel comprising of representatives from the All-India. Ophthalmological Society (AIOS), Eye Bank Association of India (EBAI), Indian Society of Cornea and Kerato refractive surgeons (ISKRS), Cornea society of India (CSI) and major governmental and private ophthalmological institutions in India.
16	Siedlecki J et al. [26]	Review (n = 21)	The novel coronavirus SARS-CoV 2, currently causing the COVID-19 pandemic, has severe implications for ophthalmology—be it because the eyes represent an important route of infection, most probably through lacrimal drainage into the nasal mucosa, or because of ocular manifestations, which, even if rather rare, can represent the first symptoms of this novel disease.

## 4. Discussion

The COVID‑19 pandemic has had an enormous impact on healthcare both by the direct mortality and morbidity associated and therefore the indirect effects of lockdown and social distancing measures to regulate. The death toll around the world has risen steadily and the challenges faced by the healthcare systems is manifold [12]. Due to the close contact with patients, procedures which generate aerosols and potential risk of presence of virus in tears there was a fear that ophthalmologists and eye‑related HCWs are at a slightly higher risk of developing the infection.

Various infection control measures are evolved within the previous couple of months to attenuate the spread of infection to both patients and health care workers. Hospitals have adopted a triage system to attenuate cross-infection. Eye Banking is an integral component of eye care services. Eye-banking activities would need strategic planning for the longer term when things normalizes. The pandemic has made the eye bankers reflect and introspect crisis that can occur and help devise a plan to handle a similar situation if it arises in the future [21].

Some of the available literature states unavailability of sufficient evidence based studies proving availability of virus in the cornea or tear of COVID affected patients [3],[19],[20],[22]. It is too early to possess evidence that SARS-CoV-2 are often transmitted by transfusion or tissue transplantation. Maybe a rapid screening test for all donors can help to rule out in the future [21].

An article by Sharma et all on Procurement and Utilization trends of eye banks in India states the precautionary measures that needs to be taken before harvesting the cornea. The harvesting can be planned after following a triage system that is used in clinics. The detailed history may be elicited from family members before collection. The collection can be restricted only to the death cases due to completely nonrelated causes like a road traffic accident, hanging, poisoning, etc., Those on ventilators of any time duration should be avoided [27].

Literature by Toro et.al and Joran D.D. et al. have stated that there is no evidence to substantiate that harvested corneal grafts from COVID-19 patients can contain SARS-CoV-2 virus and lead to a systemic infection. Although risk of transmission through corneal stromal tissue is low, it potentially exists [3]. While other literature states that we currently lack sufficient evidence to suggest that a sizable viral load presenting a significant risk to donor recipients is harbored within the corneal stroma [11].

A literature available by Amesty MA et al. on COVID-19 Disease and Ophthalmology highlights that there is need to plan a well-designed trial to rule out other ocular manifestations that may result from COVID-19 infection and to understand the transmission of the virus through the eyes [20], similar findings have also been mentioned in an editorial by Ang M, Moriyama A et.al states that more research is needed to examine the risk of ocular transmission of SARSCoV-2, both from an eye banking and healthcare provider perspective [19].

An article by Fernández-Rodríguez A et al. reviews the microbiological aspects of the COVID-19 infection and recommends that the RT-PCR is the technique of choice for detection of virus in the corpus and the sample analyzed was a pharyngeal swab, and only a few cases used samples taken from the lower airways, such as lung parenchyma swabs or lung necropsies fixed in paraffin. Study has also stated that there are very few papers that are available on the results of autopsies in cases of COVID-19 are restricted to certain organs and/or tissues, or they use minimally invasive techniques [15].

Another technical Advisory by Welsh Government states that, if COVID-19 testing on postmortem swab specimens is being considered for a suspected COVID-19 case, SARS-CoV-2 RNA may still be detected up to 3 days postmortem and possibly longer; however, sensitivity may be reduced with a longer postmortem interval, and duration of illness may need to be considered in interpreting a negative result. Advisory have also recommended that Post-mortem testing policy should be kept under review and may evolve in the response to new evidence, similar findings have also been reported in an advisory by Health department of The Republic of South Africa [17],[18].

MOHFW has published a Standard Guidelines on Safe Ophthalmology Practices in COVID-19 Scenario which outlines the preventive and response measures to be observed to minimize and avoid the spread of COVID-19 in eye care facilities. In-spite of all a general understanding was kept that the Cornea can be harvested from the COVID affected dead bodies with due precaution [28].

## 5. Conclusions

There is no proven consensus on presence of Virus in cornea. It is important to use preferred practice guidelines so that we can exert extra care to restart eye banking and do at least the emergency surgeries without facilitating disease transmission and keeping ourselves safe.
